# The impact of mind–body exercise on female breast cancer patients—a systematic review and meta-analysis of randomized controlled trials

**DOI:** 10.3389/fpubh.2025.1641075

**Published:** 2025-10-27

**Authors:** Lei Li, Yuanhada He, Xiaojuan Yang, Yang Liu

**Affiliations:** ^1^Precision Medicine Laboratory, Hohhot Maternal and Child Health Hospital, Hohhot, China; ^2^Department of Human Anatomy, School of Basic Medical Sciences, Inner Mongolia Medical University, Hohhot, China

**Keywords:** breast cancer patients, mind–body exercise, complementary and alternative therapy, systematic review, meta-analysis

## Abstract

**Objective:**

To evaluate the effects of mind–body exercise on breast cancer patients.

**Methods:**

A systematic search was conducted in the Cochrane Library, Embase, PubMed, Ovid, and Web of Science databases from inception to October 23, 2024, for randomized controlled trials (RCTs) assessing the effects of mind–body exercise on breast cancer patients. Inclusion criteria were: intervention group receiving mind–body exercises such as mindfulness or yoga; control group receiving standard care; participants aged ≥18 years with breast cancer; and outcomes including anxiety, fear of cancer recurrence (FCR), fatigue, IL-6, and 7 other indicators. Two reviewers independently screened the literature and extracted data. After assessing the methodological quality of the included studies using the Cochrane Risk of Bias tool, meta-analysis was conducted using RevMan 5.4 and Stata 15.0 software.

**Results:**

A total of 47 RCTs involving 4,537 breast cancer patients were included. Meta-analysis results showed that compared to standard care, mind–body exercise significantly improved anxiety (SMD = −0.50, 95% CI [−0.73, −0.27], *p* < 0.0001), depression (SMD = −0.43, 95% CI [−0.60, −0.26], *p* < 0.00001), insomnia (SMD = −0.40, 95% CI [−0.72, −0.07], *p* = 0.02), fatigue (SMD = −0.52, 95% CI [−0.72, −0.31], *p* < 0.00001), and FCR (SMD = −0.51, 95% CI [−0.88, −0.14], *p* = 0.007). Furthermore, it significantly reduced perceived stress (SMD = −0.65, 95% CI [−1.11, −0.20], *p* = 0.005), lowered IL-6 levels (SMD = −0.30, 95% CI [−0.56, −0.03], *p* = 0.03), and improved overall quality of life (SMD = 0.67, 95% CI [0.39, 0.95], *p* < 0.00001). Sensitivity analyses indicated that the pooled effect sizes were stable.

**Conclusion:**

Mind–body exercises can effectively alleviate anxiety, depression, and fatigue in breast cancer patients, and appear beneficial in reducing FCR. Although pooled analyses also demonstrated statistically significant improvements in perceived stress, insomnia, quality of life, and IL-6 concentrations, the strength of the current evidence is limited, and the results should be interpreted with caution.

**Systematic review registration:**

This systematic review was registered in PROSPERO under the registration number CRD42024568483. The registration details are available at: https://www.crd.york.ac.uk/PROSPERO/view/CRD42024568483.

## 1 Introduction

According to global cancer statistics in 2020, breast cancer incidence and mortality rates have surpassed lung cancer, making it the leading cause of cancer in women. In 2022, there were 2.3 million new cases of breast cancer diagnosed in women worldwide, with 670,000 deaths from the disease. It is estimated that by 2040, the number of new breast cancer diagnoses will reach 3 million, with 1 million deaths. Additionally, the risk of developing breast cancer in women increases progressively with age.

Although the majority of patients survive for more than 5 years after breast cancer diagnosis (1), the side effects of chemotherapy and radiotherapy, surgical trauma, and physical damage such as hair loss make patients more susceptible to anxiety, depression, and other negative emotions. A meta-analysis revealed that nearly 50% of female breast cancer patients experience anxiety and/or depression (2, 3), particularly those who face significant stress due to concerns about cancer recurrence. This fear further exacerbates their mental burden and may even lead to more severe psychological disorders, such as post-traumatic stress disorder (PTSD) (4). In addition, breast cancer patients often face a range of physiological issues, including sleep disturbances, pain, and fatigue (5–8). These symptoms can not only negatively affect the overall quality of life but may also interfere with treatment outcomes. Therefore, it is essential to focus not only on the clinical efficacy of breast cancer treatment but also on the psychological and physiological impacts the disease and its treatment have on patients.

As breast cancer survival rates continue to rise, patients often require longer treatment durations, which imposes a significant economic burden on both the individuals and their families. Mind–body exercise, as a complementary and alternative therapy, plays a role in regulating mental states and promoting physical health (9–11). The National Comprehensive Cancer Network (NCCN) Breast Cancer Risk Reduction Guidelines suggest that increased physical activity can reduce the risk of breast cancer to some extent (12). Another meta-analysis found that engaging in at least 150 min of moderate-intensity physical activity per week can reduce the risk of breast cancer by 9% (13). The mind–body exercise combines the benefits of resistance training and aerobic exercise, which not only enhances physical fitness but also helps regulate mood and mental states. Existing studies indicate that mind–body exercise plays a role in alleviating anxiety and depression in breast cancer patients (14, 15). However, there is still controversy over whether it can alleviate other burdens on patients, such as fatigue, pain, sleep disturbances, quality of life, and cognitive dysfunction. Moreover, there is a lack of systematic meta-analyses on the impact of mind–body exercise on inflammatory markers in breast cancer patients. Therefore, this study will explore the effects of mind–body exercises (such as mindfulness, meditation, yoga, Tai Chi, and Baduanjin) on the physical, psychological, and inflammatory markers in breast cancer patients. The findings will provide insights to support the development of non-pharmacological treatments for breast cancer, offering substantial evidence for both patients and clinicians.

## 2 Materials and methods

This paper was written following the Cochrane systematic review guidelines and the Preferred Reporting Items for Systematic Reviews and Meta-Analyses (PRISMA) standards. It has been registered on the international prospective systematic review platform (PROSPERO; registration number = CRD42024568483).

### 2.1 Search strategy

A search was conducted in Cochrane, EMBASE, Ovid, PubMed, and Web of Science from their inception until October 2024. The search strategy was based on the PICOS framework: (P) Population: breast cancer patients; (I) Intervention: mind–body exercise; (C) Comparison: standard care and appropriate rehabilitation measures; (O) Outcomes: mind–body exercise assessments in breast cancer patients; (S) Study type: randomized controlled trials. The detailed search strategy is shown in Table 1 (using PubMed as an example).

**Table 1:** Search strategy on PubMed.

#1	“Breast Neoplasms”[MeSH]
#2	(((Breast Neoplasms[Title/Abstract]) OR (Breast Neoplasm*[Title/Abstract])) OR (Breast Cancer[Title/Abstract])) OR (Breast Carcinoma[Title/Abstract])
#3	#1 OR #2
#4	“Tai Ji”[MeSH]
#5	(((((((((Tai Ji[Title/Abstract]) OR (Tai Chi[Title/Abstract])) OR (Tai Ji Quan[Title/Abstract])) OR (Tai Chi Chuan[Title/Abstract])) OR (Tai-ji[Title/Abstract])) OR (Chi, Tai[Title/Abstract])) OR (Ji Quan, Tai[Title/Abstract])) OR (Quan, Tai Ji[Title/Abstract])) OR (taijiquan[Title/Abstract])) OR (T’ai Chi[Title/Abstract])
#6	“Qigong”[MeSH]
#7	((Qigong[Title/Abstract]) OR (Ch’i Kung[Title/Abstract])) OR (Qi Gong[Title/Abstract])
#8	(Baduanjin[Title/Abstract]) OR (Eight trigrams boxing[Title/Abstract])
#9	“Meditation”[MeSH]
#10	(Meditation[Title/Abstract]) OR (Transcendental Meditation[Title/Abstract])
#11	“Yoga”[MeSH]
#12	“Yoga”[Title/Abstract]
#13	“Mind–Body Therapies”[MeSH]
#14	((Mind-Body Therapies[Title/Abstract]) OR (Mind Body Intervention[Title/Abstract])) OR (Mind-Body Exercise[Title/Abstract])
#15	#4 OR #5 OR #6 OR #7 OR #8 OR #9 OR #10 OR #11 OR #12 OR #13 OR #14
#16	“Randomized Controlled Trial”[MeSH]
#17	((Randomized Controlled Trial[Title/Abstract]) OR (random*[Title/Abstract])) OR (Controlled Clinical Trial[Title/Abstract])
#17	#16 OR #17
#18	#3 AND #15 AND #17

### 2.2 Inclusion criteria

(1) Study design: randomized controlled trials;
(2) Participants: patients aged 18 years and older, with a pathological diagnosis of breast cancer stage 0 to IV;
(3) Intervention group: patients were subjected to interventions including mindfulness, meditation, yoga, Tai Chi, and Baduanjin;
(4) Control group: patients received only standard care and appropriate rehabilitation services;
(5) Outcome measures: anxiety, depression, fatigue, sleep, quality of life, pain, stress, cognitive function, FCR, and levels of IL-6 and CRP (C-reactive protein) in the body.

### 2.3 Exclusion criteria

(1) Studies with incomplete or unreported data;
(2) Studies with duplicate publications;
(3) Non-randomized controlled trials (including animal studies, reviews, conference abstracts, and case reports).

### 2.4 Literature screening

Two researchers screened and excluded the literature using EndNote reference management software.

(1) Screening of titles to exclude duplicate studies, reviews, conference proceedings, and non-randomized controlled trials;
(2) Reviewing abstracts to further determine studies for inclusion or exclusion;
(3) Reading the full texts of the included studies to finalize their inclusion.

An independent double-blind method was employed during this process. The included studies were compared, and if the findings were consistent, they were included; if there were discrepancies, a third researcher resolved them through discussion.

### 2.5 Data extraction

Data from the included studies were extracted according to a 7-item data extraction form, with the following specific categories: (1) authors; (2) publication year; (3) country; (4) population; (5) sample size; (6) average age; and (7) details of the exercise intervention.

### 2.6 Risk of bias assessment

Bias risk assessment of the included studies were conducted according to the Cochrane Handbook for Systematic Reviews of Interventions 5.1.0, with specific criteria based on seven aspects: (1) generation of random sequence; (2) allocation concealment; (3) blinding of participants; (4) blinding of intervention providers and outcome assessors; (5) completeness of outcome data; (6) selective reporting; and (7) other sources of bias. Based on these criteria, the included studies were categorized into three levels of bias risk: high risk (five or more aspects), moderate risk (three or four aspects), and low risk (two or fewer aspects). The bias risk assessment was independently performed by two researchers, with cross-checking. In case of disagreements, a third reviewer resolved the issues (16).

### 2.7 Statistical methods

Statistical analysis was performed using Review Manager 5.4 software. As the study outcomes were continuous variables with different outcome measures, standardized mean difference (SMD) and 95% confidence intervals (CIs) were used for data analysis to minimize the impact of different measurement methods. SMD values of 0.2 to 0.5 indicate a small effect, 0.5 to 0.8 indicate a moderate effect, and values greater than 0.8 indicate a large effect (17). When the 95% CI does not include 0, the results of the meta-analysis are considered statistically significant; when the 95% CI includes 0, the results are not statistically significant. The heterogeneity of the study results was assessed using *I*^2^ and *p*-values. If *p* ≥ 0.1 and *I*^2^ ≤ 50%, a fixed-effect model was used for analysis. If *p* < 0.1 and *I*^2^ > 50%, indicating statistical heterogeneity, a random-effects model was applied and meta-regression was conducted to explore sources of heterogeneity. Additionally, sensitivity analysis was performed by sequentially excluding studies. If the results showed minimal change, it suggested that the findings were stable. Publication bias was primarily assessed using funnel plots and Egger’s linear regression method.

## 3 Results

### 3.1 Literature search and inclusion results

**Figure 1:**
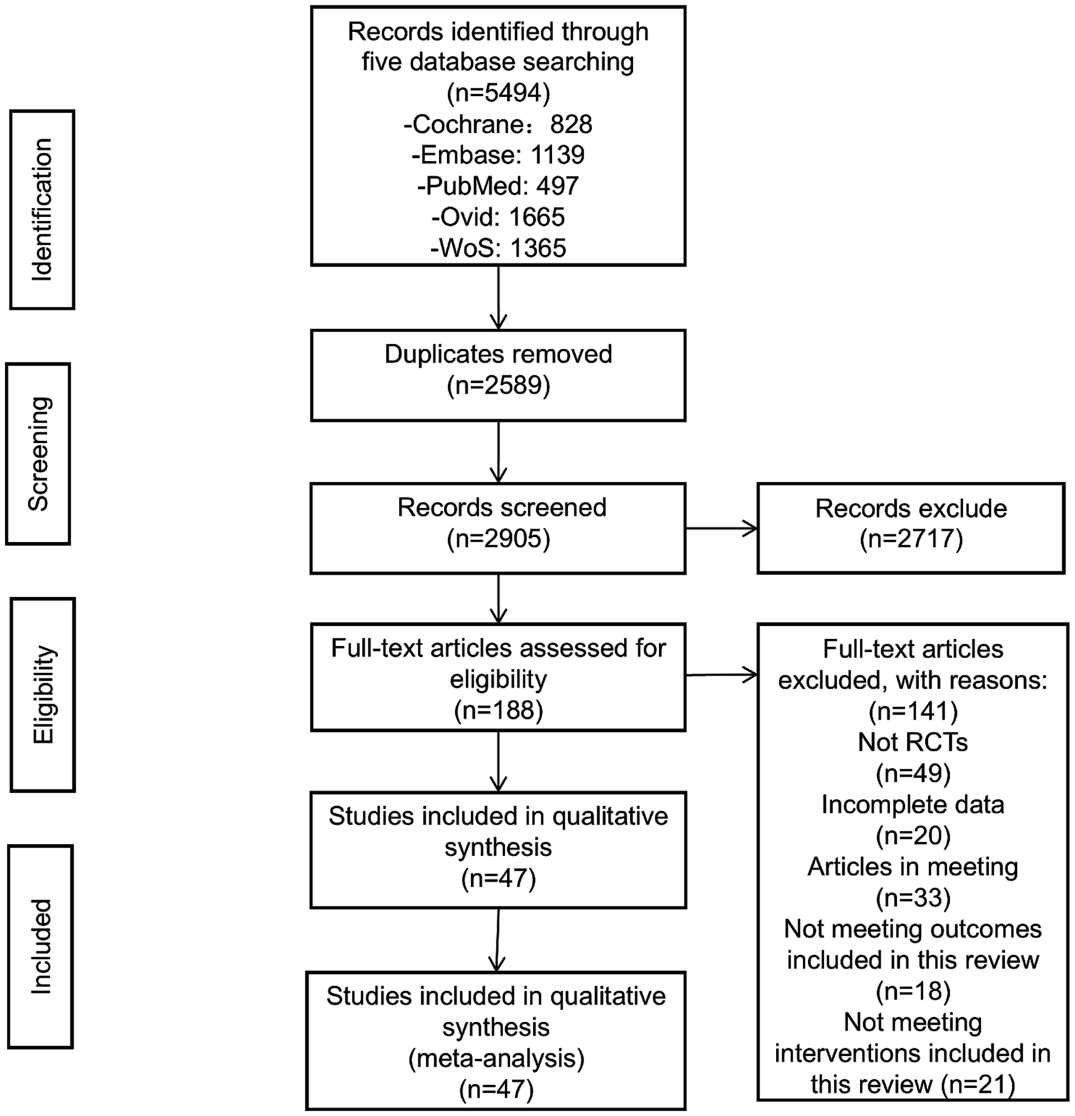
Flow diagram of literature selection.

Through the established literature search strategy, an initial retrieval identified 5,494 articles. After removing duplicates, 2,905 articles remained. Upon reviewing the titles and abstracts, 2,717 articles were excluded, leaving 188 for full-text screening. After thoroughly reading the full text, articles that were not randomized controlled trials, had incomplete data, were conference proceedings, or did not meet the intervention criteria of this review were excluded, resulting in the removal of 141 articles. Finally, 47 articles were included (18–64) (Figure 1).

### 3.2 Quality assessment of included studies

**Figure 2:**
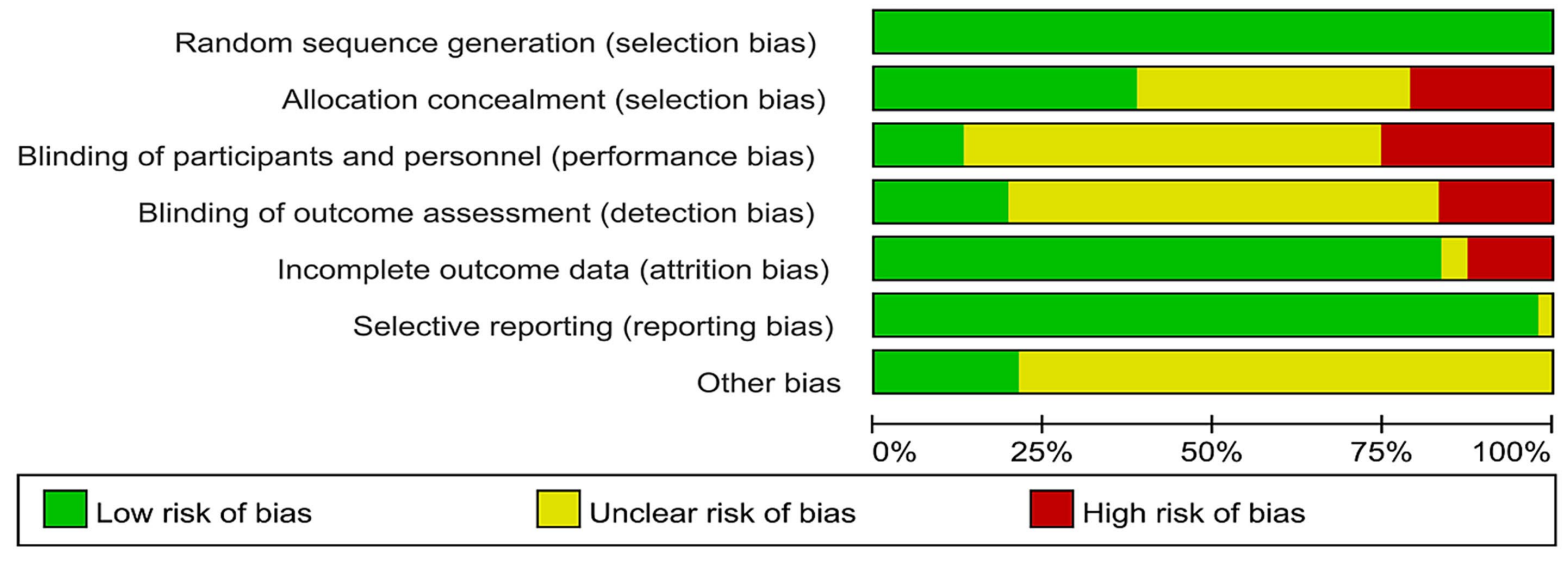
Cochrane risk of bias in the included studies.

**Figure 3:**
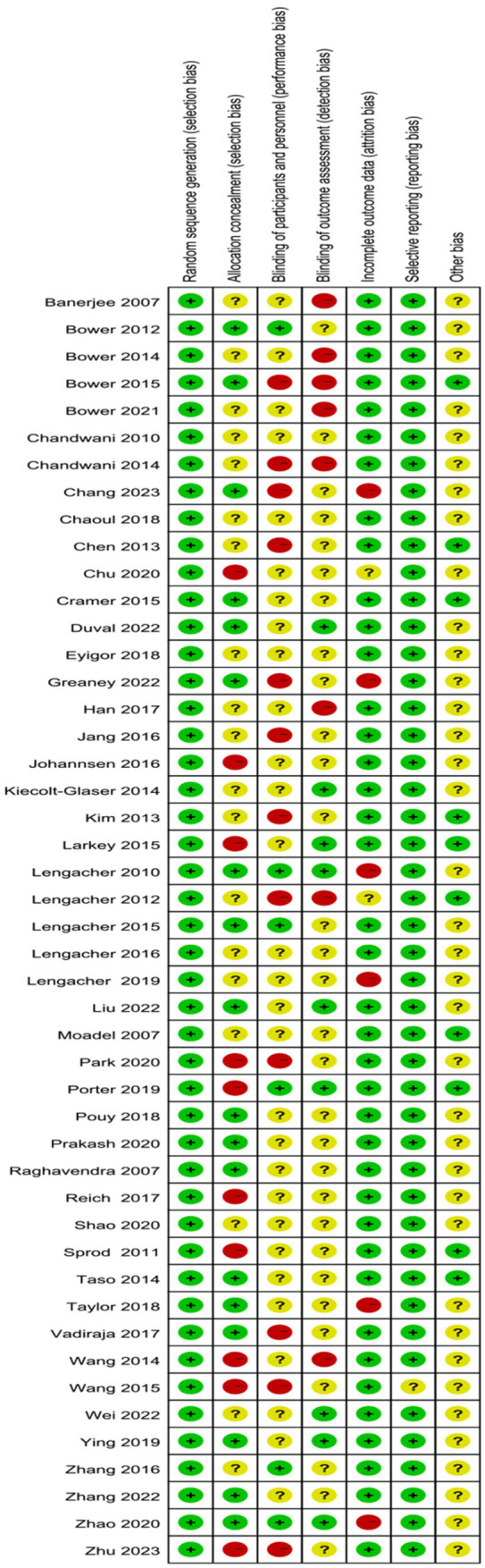
Cochrane risk of bias summary for included studies.

This study ultimately included 11 high-quality articles, 33 moderate-quality articles, and 3 low-quality articles. All included articles described the method of random grouping. Eighteen articles mentioned the method of concealing the allocation sequence; 12 articles described the process of implementing blinding, with 3 employing a double-blind method and 9 using a single-blind method. Thirty-nine articles provided complete outcome reports (Figures 2, 3).

### 3.3 Characteristics of included studies

A total of 47 RCTs were included, comprising 4,537 diagnosed breast cancer patients. The included interventions consisted of meditation training (1 study) (18), mindfulness training (18 studies) (19–36), yoga training (20 studies) (37–56), and qigong training (8 studies) (57–64), of which 24 studies were from Asia, 22 studies were from the Americas, and 1 study was from Europe, with 11 different outcome measurement scales. The details are provided in Table 2.

**Table 2:** Characteristics of the studies included in the meta-analysis.

Author	Country	Year	Population	Age (mean + SD)	Sample size (T/C)	Intervention	Control	Outcome
Kim et al. (18)	Korea	2013	Breast cancer Stage of disease 0–III	T: 48.12 (7.06) C: 46.86 (7.74)	T: 51/C: 51	Meditation training Length of intervention: 6 weeks Freq: 2 times a week Duration: 60 min	CON	Anxiety, Depression, Fatigue, QOL, Pain, Cognitive ability
Pouy et al. (19)	Iran	2018	Breast cancer Stage of disease 0–III	T: 52.12 (11.07) C: 56.14 (11.04)	T: 32/C: 34	MBSR training Length of intervention: 4 weeks Freq: 2 times a week Duration: 90 min	CON	Anxiety, Depression, QOL, Perceived stress
Lengacher et al. (27)	USA	2015	Breast cancer Stage of disease 0–III	T: 56.1 (9.1) C: 58.0 (10.2)	T: 38/C: 41	MBSR training Length of intervention: 6 weeks Freq: 120 min a week Duration: 15–45 min/day	CON	Insomnia
Duval et al. (28)	Canada	2022	Breast cancer Stage of disease NA	T: 49.20 (10.02) C: 53.47 (8.55)	T: 30/C: 30	MBSR training Length of intervention:2 weeks Freq: 8 times a week Duration: 150 min	WLC	Cognitive ability
Lengacher et al. (29)	USA	2019	Breast cancer Stage of disease 0–III	T: 56.5 (10.2) C: 57.6 (9.2)	T: 167/C: 155	MBSR training Length of intervention: 6 weeks Freq: 120 min a week Duration: 15–45 min/day	CON	IL-6 level
Zhu et al. (30)	China	2023	Breast cancer Stage of disease I–III	T: 47.96 (8.51) C: 49.78 (7.48)	T: 50/C: 51	MBSR training Length of intervention: 8 weeks Freq: 1 time a week Duration: 120 min	CON	Anxiety, Depression, QOL
Lengacher et al. (31)	USA	2010	Breast cancer Stage of disease 0–III	T + C: 57.5 (9.4)	T: 41/C: 43	MBSR training Length of intervention: 6 weeks Freq:6 times a week Duration: 15–45 min	CON	Depression, Perceived stress, FCR
Lengacher et al. (32)	USA	2016	Breast cancer Stage of disease 0–III	T: 56.5 (10.2) C: 57.6 (9.2)	T: 167/C: 155	MBSR training Length of intervention: 6 weeks Freq: 1 time a week Duration: 120 min	CON	Depression, Fatigue, Pain, QOL, FCR
Lengacher et al. (33)	USA	2012	Breast cancer Stage of disease 0–III	T + C: 58 (9.4)	T: 41/C: 43	MBSR training Length of intervention: 6 weeks Freq: 6 times a week Duration: 15–45 min	CON	Fatigue, Pain
Reich et al. (34)	USA	2017	Breast cancer Stage of disease 0–III	T + C: 56.6	T: 147/C: 152	MBSR training Length of intervention: 6 weeks Freq: 120 min a week Duration: 15–45 min/day	CON	Insomnia, Perceived stress
Zhang et al. (36)	China	2016	Breast cancer Stage of disease I–III	T: 48.6 (8.49) C: 46.0 (5.12)	T: 30/C: 30	MBSR training Length of intervention: 8 weeks Freq: 6–7 times a week Duration: 40–45 min	CON	Perceived stress
Zhao et al. (23)	China	2020	Breast cancer Stage of disease I–III	T: 52.79 (6.54) C: 53.29 (6.50)	T: 68/C: 68	MBCT training Length of intervention: 8 weeks Freq: 6 times a week Duration: 90 min	WLC	Insomnia
Johannsen et al. (24)	Denmark	2016	Breast cancer Stage of disease 0–III	T: 56.8 (9.99) C: 56.7 (8.10)	T: 67/C: 62	MBCT training Length of intervention: 8 weeks Freq: 1 time a week Duration: 30 min	CON	Pain
Park et al. (25)	Tokyo	2020	Breast cancer Stage of disease 0–III	T: 53.21 (8.4) C: 54.19 (9.27)	T: 38/C: 36	MBCT training Length of intervention: 8 weeks Freq: 120 min a week Duration: 20–45 min/day	WLC	Anxiety, Depression, Fatigue, QOL, FCR
Chu et al. (26)	China	2020	Breast cancer Stage of disease 0–III	T: 54.6 (5.7) C: 54.9 (6.3)	T: 42/C: 42	MBCT training Length of intervention: 8 weeks Freq: 120 min a week Duration: 20–45 min/day	CON	Anxiety, Depression, Fatigue, QOL, FCR
Jang et al. (21)	Korea	2016	Breast cancer Stage of disease 0–III	T: 51.75 (5.32) C: 51.42 (6.33)	T: 12/C: 12	MBAT training Length of intervention: 12 weeks Freq: NA Duration: 45 min	CON	QOL, Cognitive ability
Bower et al. (20)	USA	2015	Breast cancer Stage of disease 0–III	T: 46.1 (7.9) C: 47.7 (7.1)	T: 39/C: 32	MAPs training Length of intervention: 6 weeks Freq: 6 times a week Duration: 20 min	CON	Fatigue, Insomnia, Pain, FCR, IL-6 level, CRP level
Bower et al. (35)	USA	2021	Breast cancer Stage of disease 0–III	T: 44.5 (7.7) C: 45.9 (5.6)	T: 85/C: 81	MAPs training Length of intervention: 6 weeks Freq: 120 min a week Duration: NA	WLC	Depression, Fatigue, Insomnia
Shao et al. (22)	China	2020	Breast cancer Stage of disease I–IV	T: 40.3 (7.0) C: 44.4 (8.2)	T: 72/C: 72	MBIs training Length of intervention: 6 weeks Freq: 5 times a week Duration: 20 min	CON	Anxiety, Depression
Taylor et al. (37)	USA	2018	Breast cancer Stage of disease NA	T: 54.9 (8.8) C: 52.6 (8.2)	T: 14/C: 12	Yoga training Length of intervention: 8 weeks Freq: 1 time a week Duration: 75 min	WLC	Depression, Fatigue, Insomnia, Perceived stress
Wang et al. (38)	China	2014	Breast cancer Stage of disease NA	T + C: 39 (10.5)	T: 40/C: 42	Yoga training Length of intervention: 4 months Freq: 4 times a week Duration: 50 min	CON	Fatigue
Taso et al. (39)	Taiwan	2014	Breast cancer Stage of disease I–III	T + C: 49.27 (10.23)	T: 30/C: 30	Yoga training Length of intervention: 8 weeks Freq: 2 times a week Duration: 60 min	CON	Fatigue
Wang et al. (40)	China	2015	Breast cancer Stage of disease 0–IV	T + C: 39 (10.5)	T: 40/C: 42	Yoga training Length of intervention: 4 months Freq: 4 times a week Duration: 50 min	CON	QOL
Raghavendra et al. (41)	India	2007	Breast cancer Stage of disease II–III	T + C: 50 (10)	T: 28/C: 34	Yoga training Length of intervention: After fourth chemotherapy Freq: 6 times a week Duration: 60 min	CON	Depression, QOL
Liu et al. (42)	China	2022	Breast cancer Stage of disease I–II	T + C: 48 (2.25)	T: 68/C: 68	Mindfulness yoga training Length of intervention: 8 weeks Freq: 90 min a week Duration: NA	CON	Anxiety, Depression, Fatigue, QOL,
Prakash et al. (43)	India	2020	Breast cancer Stage of disease NA	NA	T: 48/C: 52	Yoga training Length of intervention: 3 weeks Freq: 2 times a week Duration: 60 min	CON	QOL
Chandwani et al. (44)	USA	2014	Breast cancer Stage of disease 0–III	T: 52.38 (1.35) C: 52.11 (1.34)	T: 53/C: 54	Yoga training Length of intervention: 6 weeks Freq: 3 times a week Duration: 60 min	CON	Fatigue, Insomnia
Chandwani et al. (45)	USA	2010	Breast cancer Stage of disease 0–III	T: 51.39 (7.97) C: 54.02 (9.96)	T: 30/C: 31	Yoga training Length of intervention: 6 weeks Freq: 2 times a week Duration: 60 min	WLC	Depression, Fatigue, Insomnia, Pain
Moadel et al. (46)	USA	2007	Breast cancer Stage of disease I–IV	T: 55.11 (10.07) C: 54.23 (9.81)	T: 84/C: 44	Yoga training Length of intervention: 12 weeks Freq: 90 min a week Duration: NA	CON	Fatigue, QOL
Cramer et al. (47)	USA	2015	Breast cancer Stage of disease I–III	T: 48.3 (4.8) C: 50.0 (6.7)	T: 19/C: 21	Yoga and meditation training Length of intervention: 12 weeks Freq: 90 min a week Duration: NA	CON	Anxiety, Depression, Fatigue, QOL
Banerjee et al. (48)	India	2007	Breast cancer Stage of disease II–III	T: 47 (1.1) C: 43 (1.5)	T: 35/C: 23	Yoga training Length of intervention: 6 weeks Freq: NA Duration: 90 min	CON	Perceived stress
Kiecolt-Glaser et al. (49)	USA	2014	Breast cancer Stage of disease 0–III	T: 51.8 (9.8) C: 51.3 (8.7)	T: 100/C: 100	Hatha yoga training Length of intervention: 12 weeks Freq: 2 times a week Duration: 90 min	CON	Depression, Fatigue, IL-6 level
Porter et al. (50)	USA	2019	Breast cancer Stage of disease IV	T: 56.3 (11.6) C: 59.4 (11.3)	T: 43/C: 20	Mindful yoga training Length of intervention: 8 weeks Freq: 8 times a week Duration: 120 min	SSG	Anxiety, Depression, Fatigue, Insomnia, pain
Greaney et al. (51)	USA	2022	Breast cancer Stage of disease I–III	T: 53.2 (10.1) C: 49.9 (13.5)	T: 15/C: 15	Yoga training Length of intervention: 12–20 weeks Freq: 3 times a week Duration: 30 min	CON	Fatigue, QOL, CRP level
Chaoul et al. (52)	USA	2018	Breast cancer Stage of disease I–III	T: 49.5 (9.8) C: 49 (10.1)	T: 74/C: 85	Tibetan yoga training Length of intervention: 4–12 weeks Freq: 2 times a week Duration: 75–90 min	CON	Fatigue, Insomnia
Bower et al. (53)	USA	2014	Breast cancer Stage of disease 0–II	T + C: 54 (5.4)	T: 16/C: 15	Yoga training Length of intervention: 12 weeks Freq: NA Duration: NA	CON	IL-6 level, CRP level
Eyigor et al. (54)	Turkey	2018	Breast cancer Stage of disease NA	T: 52.3 (9.5) C: 51.5 (7.3)	T: 22/C: 20	Yoga training Length of intervention: 10 weeks Freq: 2 times a week Duration: 60 min	CON	Depression, QOL
Bower et al. (55)	USA	2012	Breast cancer Stage of disease 0–II	T: 54.4 (5.7) C: 53.3 (4.9)	T: 16/C: 15	Yoga training Length of intervention: 12 weeks Freq: 2 times a week Duration: 90 min	Health Education	Depression, Fatigue, Insomnia, Perceived stress
Vadiraja et al. (56)	India	2017	Breast cancer Stage of disease IV	T + C: 50.54 (8.53)	T: 46/C: 45	Yoga training Length of intervention: 3 months Freq: 3 times a week Duration: 60 min	CON	Fatigue, Perceived stress
Han et al. (57)	China	2017	Breast cancer Stage of disease I–III	T: 46.23 (8.89) C: 47.83 (8.04)	T: 32/C: 32	Baduanjin training Length of intervention: 3 months Freq: 5 times a week Duration: 20 min	CON	Anxiety
Wei et al. (58)	China	2022	Breast cancer Stage of disease I–III	T: 52 (4.25) C: 55 (3)	T: 35/C: 35	Baduanjin training Length of intervention: 3 months Freq: 5 times a week Duration: 30 min	CON	Anxiety, Depression, Fatigue, QOL, Cognitive ability
Ying et al. (59)	China	2019	Breast cancer Stage of disease I–III	T + C: 54.09 (7.76)	T: 46/C: 40	Baduanjin training Length of intervention: 6 months Freq: 3–4 times a week Duration: 20 min	CON	Anxiety, Depression
Chen et al. (60)	China	2013	Breast cancer Stage of disease 0–III	T: 45.3 ± 6.3\u00B0C: 44.7 ± 9.7	T: 49/C: 47	Guo Lin Qigong training Length of intervention: 5–6 weeks Freq: 4 times a week Duration: 31–37 min	CON	Depression, Fatigue, Insomnia, QOL
Chang et al. (61)	China	2023	Breast cancer Stage of disease II–III	T: 51.91 (10.51) C: 52.77 (8.53)	T: 30/C: 30	Chan-Chuang Qigong training Length of intervention: 15 weeks Freq: 5 times a week Duration: 35 min	CON	Fatigue, Insomnia, Pain, Cognitive ability
Zhang et al. (62)	China	2022	Breast cancer Stage of disease I–III	T: 47.79 (5.14) C: 47.20 (7.65)	T: 29/C: 30	Mindfulness-based Tai Chi training Length of intervention: 8 weeks Freq: 2 times a week Duration: 60 min	WLC	Anxiety, Perceived stress
Larkey et al. (63)	USA	2015	Breast cancer Stage of disease 0–III	T: 57.7 (8.94) C: 59.8 (8.93)	T: 42/C: 45	Tai Chi training Length of intervention: 12 weeks Freq: 5 times a week Duration: 30 min	SQC	Depression, Fatigue, Insomnia
Sprod et al. (64)	USA	2011	Breast cancer Stage of disease 0–IIIb	T: 54.33 (3.55) C: 52.70 (2.11)	T: 9/C: 10	Tai Chi training Length of intervention: 12 weeks Freq: 3 times a week Duration: 60 min	SST	QOL, IL-6 level

CON, Control Group with Routine Care (no exercise); WLC, Wait-List Control Group; SST, Standard Support Therapy Control; SQC, Sham Qigong; SSG, Social Support Group; T, Experimental Group; C, Control Group; T + C, The ages of the experimental and control groups were not reported separately in the study, only the overall age was reported; QOL, Quality of Life; FCR, Fear of Cancer Recurrence.

### 3.4 Meta-analysis results

#### 3.4.1 Psychological status

##### 3.4.1.1 Anxiety

**Figure 4:**
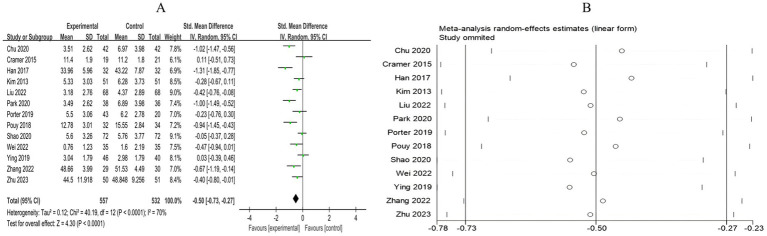
**(A)** Forest plot of the effect of mind–body exercise on anxiety in breast cancer patients. **(B)** Sensitivity analysis of anxiety levels.

A total of 13 RCTs included 1,089 breast cancer patients to compare the differences in anxiety levels between the mind–body exercise group and the control group. Significant heterogeneity was observed between the studies (*I*^2^ = 70%), and a random-effects model was used for the analysis. The results indicated that mind–body exercise significantly improved the anxiety levels of breast cancer patients (SMD = −0.50, 95% CI [−0.73, −0.27], *p* < 0.0001) (Figure 4A).

##### 3.4.1.2 Depression

**Figure 5:**
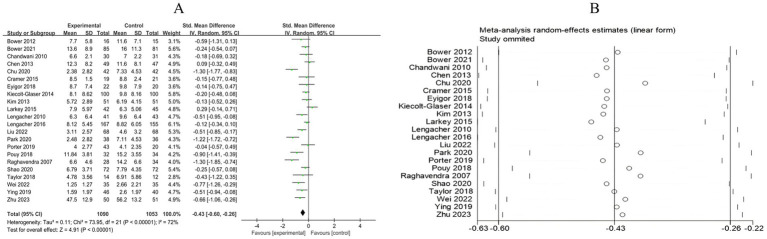
**(A)** Forest plot of the effect of mind–body exercise on depression in breast cancer patients. **(B)** Sensitivity analysis of depression levels.

A total of 22 RCTs included 2,143 breast cancer patients to compare the differences in depression levels between the mind–body exercise group and the control group. Significant heterogeneity was observed between the studies (*I*^2^ = 72%), and a random-effects model was used for the analysis. The results indicated that the mind–body exercise group significantly improved the depression levels of breast cancer patients (SMD = −0.43, 95% CI [−0.60, −0.26], *p* < 0.00001) (Figure 5A).

##### 3.4.1.3 Perceived stress

**Figure 6:**
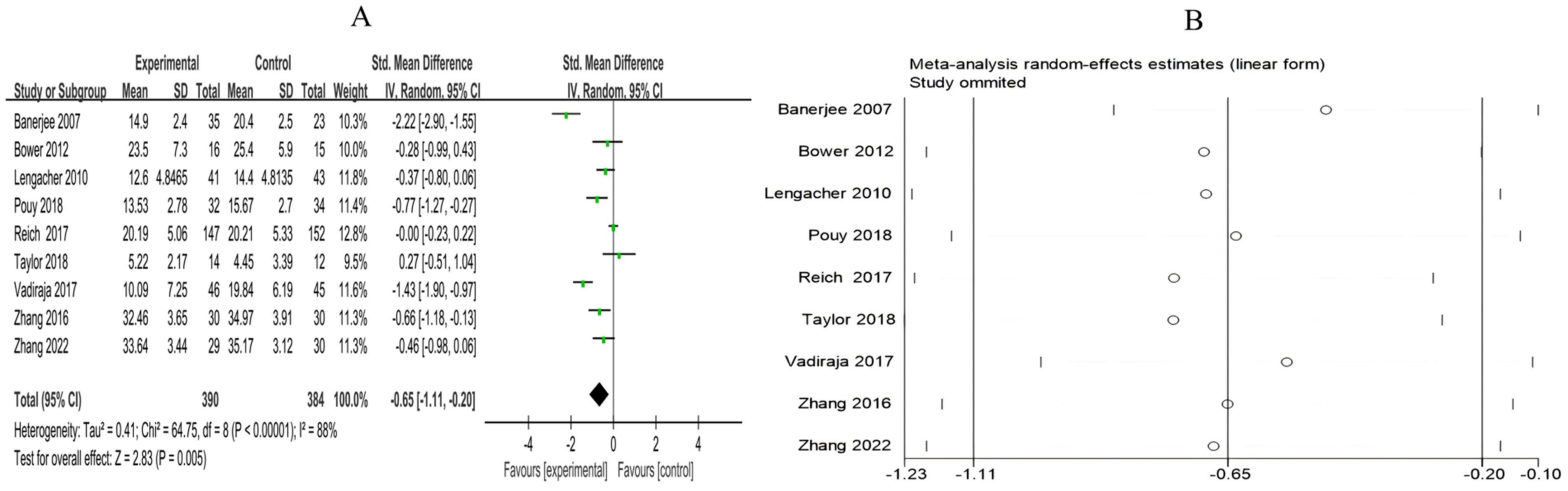
**(A)** Forest plot of the effect of mind–body exercise on perceived stress in breast cancer patients. **(B)** Sensitivity analysis of perceived stress levels.

A total of 9 RCTs included 774 breast cancer patients to examine the differences in stress levels between the mind–body exercise group and the control group. Significant heterogeneity was observed between the studies (*I*^2^ = 88%), and a random-effects model was used for the analysis. The results indicated that mind–body exercise significantly improved the stress levels of breast cancer patients (SMD = −0.65, 95% CI [−1.11, −0.20], *p* = 0.005) (Figure 6A).

##### 3.4.1.4 FCR

**Figure 7:**
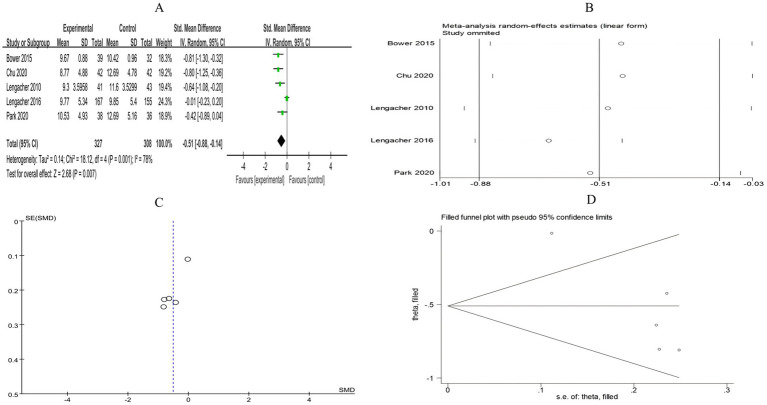
**(A)** Forest plot of the effect of mind–body exercise on FCR in breast cancer patients. **(B)** Sensitivity analysis of FCR levels. **(C)** Funnel plot of FCR. **(D)** Funnel plot of FCR using Trim-and-Fill method.

A total of 5 RCTs included 635 breast cancer patients to compare the differences in cancer recurrence fear between the mind–body exercise group and the control group. Significant heterogeneity was observed between the studies (*I*^2^ = 78%), and a random-effects model was used for the analysis. The results indicated that mind–body exercise significantly alleviated the fear of breast cancer recurrence in patients (SMD = −0.51, 95% CI [−0.88, −0.14], *p* = 0.007) (Figure 7A).

#### 3.4.2 Function and health

##### 3.4.2.1 Insomnia

**Figure 8:**
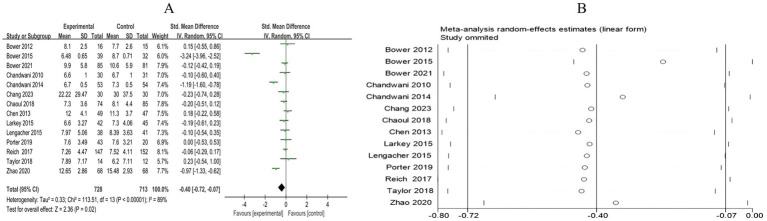
**(A)** Forest plot of the effect of mind–body exercise on insomnia in breast cancer patients. **(B)** Sensitivity analysis of Insomnia levels.

A total of 14 RCTs included 1,441 breast cancer patients to compare the differences in insomnia between the mind–body exercise group and the control group. Significant heterogeneity was observed between the studies (*I*^2^ = 89%), and a random-effects model was used for the analysis. The results indicated that, compared to the control group, the mind–body exercise group better alleviated insomnia in breast cancer patients (SMD = −0.4, 95% CI [−0.72, −0.07], *p* = 0.02) (Figure 8A).

##### 3.4.2.2 Fatigue

**Figure 9:**
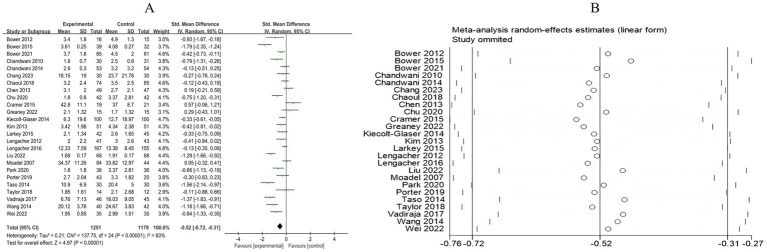
**(A)** Forest plot of the effect of mind–body exercise on fatigue in breast cancer patients. **(B)** Sensitivity analysis of fatigue levels.

A total of 25 RCTs included 2,430 breast cancer patients to examine the impact of mind–body exercise on fatigue levels in patients. Significant heterogeneity was observed between the studies (*I*^2^ = 83%), and a random-effects model was used for the analysis. The results indicated that mind–body exercise significantly improved fatigue in breast cancer patients (SMD = −0.52, 95% CI [−0.72, −0.31], *p* < 0.00001) (Figure 9A).

##### 3.4.2.3 Cognitive function

**Figure 10:**
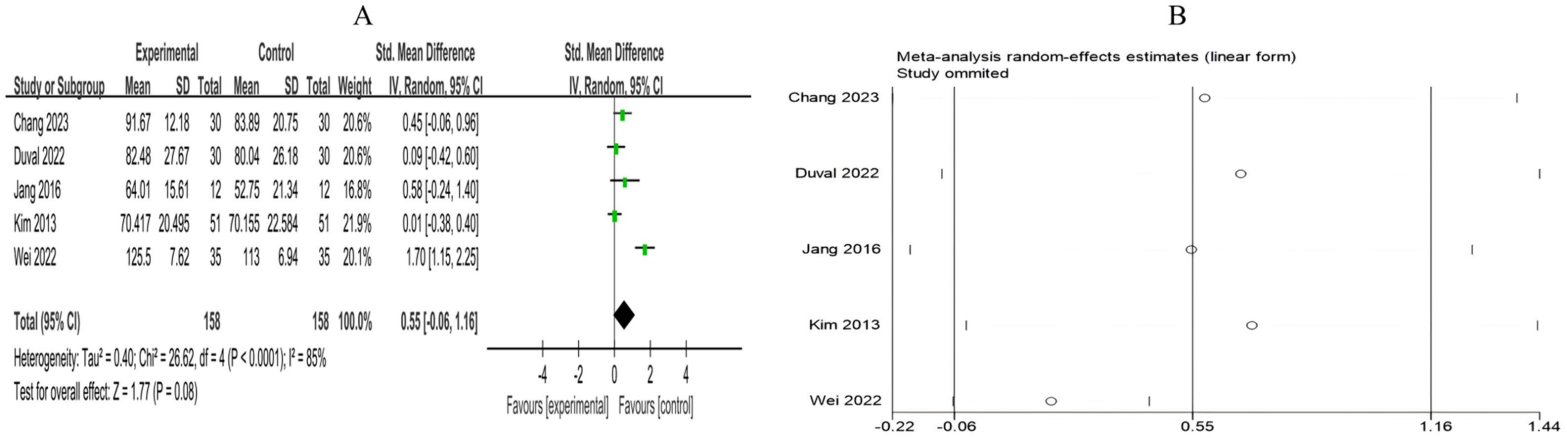
**(A)** Forest plot of the effect of mind–body exercise on cognitive function in breast cancer patients. **(B)** Sensitivity analysis of cognitive function levels.

A total of 5 RCTs included 316 breast cancer patients to examine the impact of mind–body exercise on cognitive function in patients. Significant heterogeneity was observed between the studies (*I*^2^ = 85%), and a random-effects model was used for the analysis. The results indicated that mind–body exercise had no significant effect on cognitive function in breast cancer patients (SMD = 0.55, 95% CI [−0.06, 1.16], *p* = 0.08) (Figure 10A).

##### 3.4.2.4 Pain

**Figure 11:**
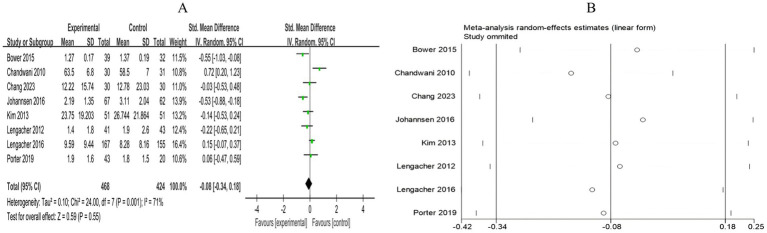
**(A)** Forest plot of the effect of mind–body exercise on pain in breast cancer patients. **(B)** Sensitivity analysis of pain levels.

A total of 8 RCTs included 892 breast cancer patients to compare the differences in pain levels between the mind–body exercise group and the control group. Significant heterogeneity was observed between the studies (*I*^2^ = 71%), and a random-effects model was used for the analysis. The results indicated that mind–body exercise had no significant effect on pain levels in breast cancer patients (SMD = −0.08, 95% CI [−0.34, 0.18], *p* = 0.55) (Figure 11A).

##### 3.4.2.5 Quality of life

**Figure 12:**
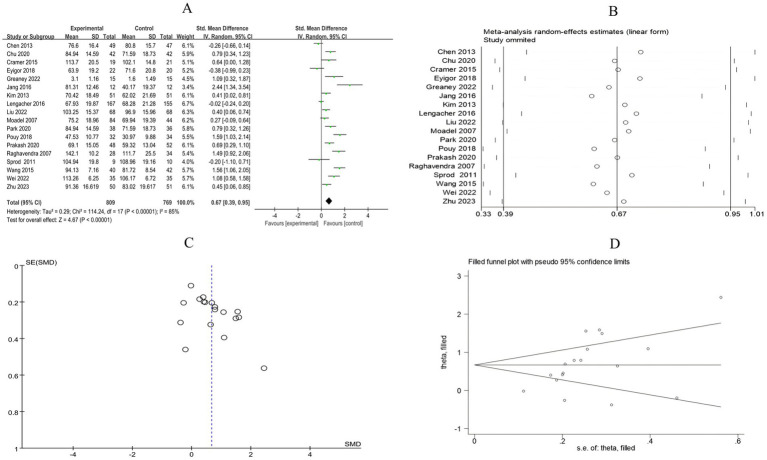
**(A)** Forest plot of the effect of mind–body exercise on QOL in breast cancer patients. **(B)**. Sensitivity analysis of QOL levels. **(C)** Funnel plot of QOL. **(D)** Funnel plot of QOL using Trim-and-Fill method.

A total of 18 RCTs included 1,578 breast cancer patients to compare the differences in quality of life between the mind–body exercise group and the control group. Significant heterogeneity was observed between the studies (*I*^2^ = 85%), and a random-effects model was used for the analysis. The results indicated that mind–body exercise significantly improved the quality of life in breast cancer patients (SMD = 0.67, 95% CI [0.39, 0.95], *p* < 0.00001) (Figure 12A).

#### 3.4.3 Biomarkers

##### 3.4.3.1 IL-6 levels

**Figure 13:**
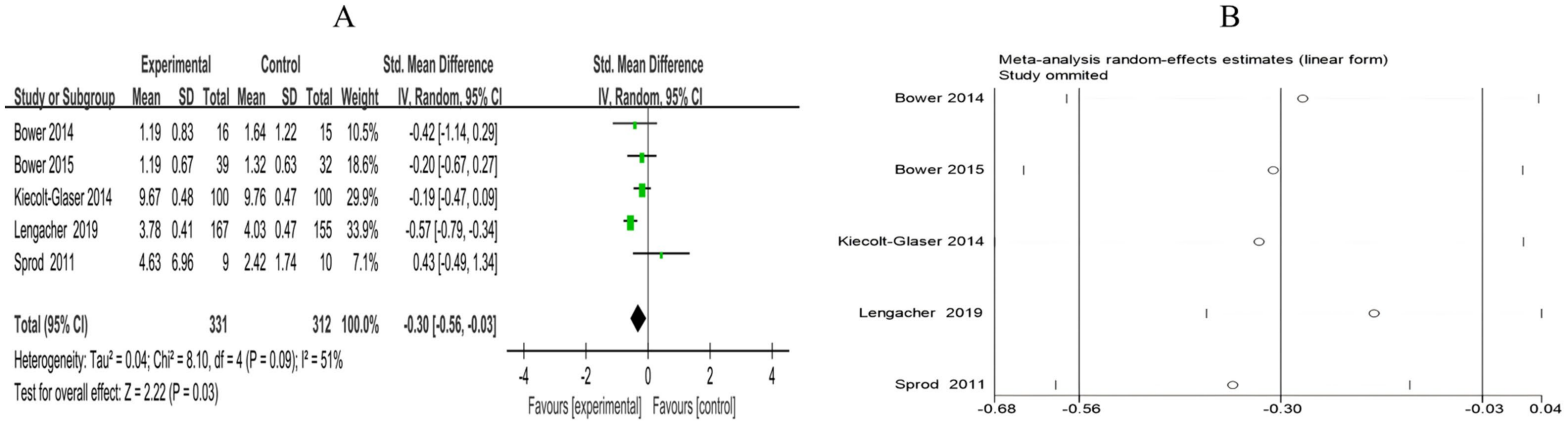
**(A)** Forest plot of the effect of mind–body exercise on IL-6 levels in breast cancer patients. **(B)** Sensitivity analysis of IL-6 levels.

A total of 5 RCTs included 643 breast cancer patients to compare the differences in IL-6 levels between the mind–body exercise group and the control group. Moderate heterogeneity was observed between the studies (*I*^2^ = 51%), and a random-effects model was used for the analysis. The results indicated that mind–body exercise significantly reduced the IL-6 levels in breast cancer patients (SMD = −0.30, 95% CI [−0.56, −0.03], *p* = 0.03) (Figure 13A).

##### 3.4.3.2 Reactive protein (CRP)

**Figure 14:**
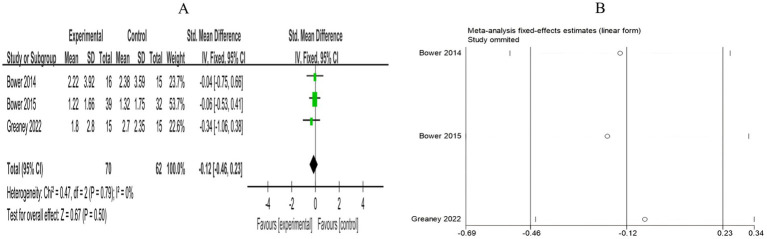
**(A)** Forest plot of the effect of mind–body exercise on CRP levels in breast cancer patients. **(B)** Sensitivity analysis of CRP levels.

A total of 3 RCTs included 132 breast cancer patients to compare the differences in CRP levels between the mind–body exercise group and the control group. No heterogeneity was observed between the studies (*I*^2^ = 0%), and a fixed-effects model was used for the analysis. The results indicated that the combined effect size was SMD = −0.12, 95% CI [−0.46, 0.23], *p* = 0.50, indicating that, compared to the control group, mind–body exercise had no significant effect on CRP levels in breast cancer patients (Figure 14A).

The summary of the meta-analysis results is provided in detail in Table 3.

**Table 3:** Effects of mind–body exercises on outcome measures in breast cancer patients.

Outcome	n		Effect size SMD	95% confidence interval		*I*^2^(%)	Df	*Z*	*P*
	Experimental	Control		Lower limit	Upper limit				
Anxiety	557	532	−0.50	−0.73	−0.27	70%	12	4.30	*P* < 0.0001
Depression	1,090	1,053	−0.43	−0.60	−0.26	72%	21	4.91	*P* < 0.00001
Fatigue	1,251	1,179	−0.52	−0.72	−0.31	83%	24	4.97	*P* < 0.00001
QOL	809	769	0.67	0.39	0.95	85%	17	4.67	*P* < 0.00001
Pain	468	424	−0.08	−0.34	0.18	71%	7	0.59	*p* = 0.05
Cognitive function	158	158	0.55	−0.06	1.16	85%	4	1.77	*P* = 0.08
Perceived stress	390	384	−0.65	−1.11	−0.20	88%	8	2.83	*P* = 0.005
FCR	327	308	−0.51	−0.88	−0.14	78%	4	2.68	*P* = 0.007
Insomnia	728	731	−0.40	−0.72	−0.07	89%	13	2.36	*P* = 0.02
IL-6 level	331	312	−0.30	−0.56	−0.03	51%	4	2.22	*P* = 0.03
CRP level	70	62	−0.12	−0.46	0.23	0%	2	0.67	*P* = 0.50

QOL, Quality of Life; FCR, Fear of Cancer Recurrence.

### 3.5 Meta-regression results

To further explore the sources of heterogeneity among the included studies, we conducted a meta-regression analysis using intervention duration, region, cancer stage, patient age, and intervention type as independent variables, and the SMD of each outcome measure as the dependent variable.

The results indicated that intervention duration was an important source of heterogeneity for multiple outcomes. Specifically, the effect sizes for anxiety (*β* = 0.061, *p* = 0.040) and quality of life (*β* = 0.150, *p* = 0.005) were positively associated with intervention duration, while those for pain (*β* = −0.178, *p* = 0.005) and cognitive function (*β* = −0.225, *p* = 0.007) were negatively associated. Regional factors significantly influenced heterogeneity in depression (*β* = 0.526, *p* = 0.001), cognitive function (*β* = −2.765, *p* = 0.001), and quality of life (*β* = −0.990, *p* = 0.004), indicating clear differences across regions. Intervention type significantly affected heterogeneity in pain (*β* = 0.881, *p* = 0.004) and depression (*β* = 0.273, *p* = 0.016). Age also had significant effects on depression (*β* = −0.034, *p* = 0.040) and cognitive function (*β* = 0.506, *p* < 0.001), with the improvement in depression showing a negative correlation with age.

For other outcomes, such as fatigue, perceived stress, insomnia, and IL-6, heterogeneity could not be explained by the variables examined in this study (all *p* > 0.05), suggesting that these outcomes may be influenced by other unmeasured factors.” The full meta-regression results are included in the Supplementary material.

### 3.6 Sensitivity analysis and publication bias

Sensitivity analysis was conducted for all outcome measures (Figures 4B–14B). The sensitivity analysis was performed using a leave-one-out approach. The results showed that the direction of effect sizes remained unchanged after excluding individual studies, indicating that the results were relatively stable. Publication bias was assessed, and Egger’s test showed no significant bias (*p* > 0.05), with the funnel plot displaying symmetry, indicating no publication bias. The funnel plots and Egger’s test showed symmetry for most outcome measures, with *p* > 0.05. Only cancer recurrence fear (Figure 7C) and quality of life (Figure 12C) showed *p* < 0.05 in Egger’s test (Table 4). We attempted trim-and-fill analysis (Figures 7D, 12D) but did not find any significant impact on the results.

**Table 4:** Results of publication bias Egger’s linear regression test.

Variable	*b*	SE	*t*	95%CI		*P*
Anxiety	−3.917	2.606	−1.50	−9.653	1.819	0.161
Depression	−2.380	1.237	−1.92	−4.960	0.199	0.069
Fatigue	−2.368	1.541	−1.54	−5.556	0.821	0.138
QOL*	4.086	1.477	2.77	0.956	7.227	0.014
Pain	−0.890	2.224	−0.40	−6.333	4.553	0.703
Cognitive function	4.703	5.375	0.87	−12.404	21.809	0.870
Perceived stress	−3.684	2.227	−1.65	−8.950	1.583	0.142
FCR*	−5.337	1.020	−5.23	−8.582	−2.091	0.014
Insomnia	−2.360	2.413	−0.98	−7.618	2.897	0.347
IL-6 level	3.001	2.742	1.09	−4.613	10.614	0.335
CRP level	−1.100	1.568	−0.70	−21.027	18.826	0.610

*Egger’s test results *P* < 0.05, QOL, Quality of Life; FCR, Fear of Cancer Recurrence.

## 4 Discussion

Anxiety and depression are common psychological issues among breast cancer patients and are significant factors contributing to higher mortality and cancer recurrence rates (65). This study is consistent with previous meta-analyses, which demonstrated that mind–body exercise significantly alleviates anxiety and depression in patients (62, 66). The heterogeneity of the combined effect sizes for anxiety and depression was moderate, with sensitivity analysis showing stable results. Meta-regression analysis suggested that the heterogeneity may be attributable to differences in intervention type, intervention duration, geographic region, and patient age. A relatively large number of clinical RCTs were included in this study, some of which were recently published, further confirming the effectiveness of mind–body exercise in addressing these common negative emotions. An increasing body of research shows that stress is closely related to various psychological and physiological problems in breast cancer patients (67–69). Stress typically refers to external circumstances or stimuli in the environment that may impact an individual’s psychological or physiological state. Perceived stress, on the other hand, focuses on an individual’s subjective experience of these stressors, emphasizing the intensity of the perceived stress. Breast cancer patients often experience significant psychological stress due to concerns about cancer recurrence. If this stress persists, it may lead to more severe mental health disorders, such as post-traumatic stress disorder (PTSD) (70–72). Therefore, perceived stress is a more accurate reflection of the actual psychological burden on breast cancer patients compared to general stress. This study demonstrates that mind–body exercise has a moderate effect in reducing perceived stress among breast cancer patients. However, due to the high heterogeneity across studies, these conclusions should be interpreted with caution. To further explore the impact of mind–body exercise on the psychological state of breast cancer patients, we included cancer recurrence fear as a research indicator and incorporated 5 RCTs. The results indicated that mind–body exercise had a moderate effect on alleviating cancer recurrence fear in patients. Sensitivity analysis confirmed the robustness of this effect, supporting the efficacy of the intervention. The Analysis of patients’ perceived stress and FCR indicates that mind–body exercise may have a potential positive effect in reducing psychological stress and enhancing psychological resilience.

Sleep disorders and fatigue are among the most common and distressing symptoms experienced by cancer patients (73). Approximately 30–75% of newly diagnosed or recently treated patients report sleep problems (73, 74), while 70–80% of patients suffer from cancer-related fatigue (75). This study included 14 RCTs with 1,441 patients and 25 RCTs with 2,430 patients, investigating the effects of mind–body exercise on insomnia and cancer-related fatigue in breast cancer patients. The results indicate that mind–body exercise has a mild effect on improving patients’ insomnia (SMD = −0.40, *p* = 0.02), showing a certain marginal effect, but had a more significant effect on alleviating fatigue (SMD = −0.52, *p* < 0.00001). This may be because insomnia is influenced not only by physiological factors but also by environmental factors, psychological states, and side effects caused by treatment. Mind–body exercise may have a short-term positive impact on sleep by improving the patient’s mindset and promoting physical relaxation. However, for long-term sleep issues, it is recommended to combine mind–body exercise with other therapeutic approaches to achieve better outcomes. Furthermore, this study found that mind–body exercise significantly improved overall quality of life in breast cancer patients (SMD = 0.67, *p* < 0.00001). Although the pooled effects for insomnia and quality of life showed considerable heterogeneity, the overall trend indicates that mind–body exercise can serve as an adjunctive intervention to improve both physical and psychological well-being in breast cancer patients.

However, this study did not find that mind–body exercise had a significant effect on cognitive function, pain, or C-reactive protein levels in breast cancer patients. This may be related to factors such as the duration of the intervention, individual differences, and floor effects due to insufficient sample size. Currently, research on the impact of mind–body exercise on cognitive dysfunction in breast cancer patients is limited. A retrospective analysis showed that 32% of studies found physical exercise helped improve cancer-related cognitive function, 2.1% showed no significant effect, and 66% did not draw definitive conclusions (76). Among the 5 RCTs included in this study, after excluding the study by Wei et al. (58) with high heterogeneity (I^2^ = 0%), a fixed-effects model was used for re-analysis. The results showed a trend of improvement in cognitive function following mind–body exercise interventions, but the effect was not statistically significant. Additionally, evidence regarding the positive impact of exercise on cancer-related pain is weak (77). This study included 8 RCTs related to breast cancer pain, but did not demonstrate a significant alleviating effect of mind–body exercise on pain. Research has shown that exercise is associated with a reduction in the levels of several pro-inflammatory cytokines (78, 79). In our meta-analysis, mind–body exercise significantly reduced IL-6 levels but did not show a significant impact on CRP levels.

## 5 Strengths and limitations

A key strength of this meta-analysis is its inclusion of a large sample size, which included 47 clinical RCTs and 4,537 patients, with a comprehensive search and analysis conducted across five databases. It systematically explored the effects of mind–body exercise on both psychological and physical functioning in breast cancer patients, providing updated and comprehensive evidence for non-pharmacological treatments for breast cancer patients.

However, this study has several limitations. First, among the 47 included studies, only 11 were rated as high-quality, while 33 were of moderate quality and 3 were low-quality, resulting in an overall limited study quality that may reduce the strength and applicability of the clinical evidence. Second, most studies did not implement blinding, increasing the risk of performance and detection bias and thus partially affecting the objectivity of the results. Third, for outcomes such as anxiety and depression, the included studies used different assessment scales to measure the same outcomes. Although we converted effect sizes obtained from different scales into SMD to provide a dimensionless and comparable metric, differences in sensitivity and scoring characteristics among the scales may still introduce methodological heterogeneity, potentially limiting the precision of direct comparisons and the robustness of pooled interpretations. Fourth, the term “mind–body exercise” encompasses various forms, including yoga, Qigong, and Tai Chi. While these interventions share common theoretical foundations and core mechanisms, they differ in intervention type, frequency, and target populations. Although pooled analyses help summarize overall trends, they may introduce significant clinical heterogeneity, and some conclusions should be interpreted with caution. Fifth, most studies did not report participants’ adherence to the interventions, making it difficult to accurately assess the real-world effectiveness of these interventions, which may affect the reliability of the results. Sixth, this study focused on the immediate effects of mind–body exercise and lacked evaluations of long-term outcomes, limiting comprehensive assessment of its sustainable benefits. Future studies should extend follow-up periods to verify the durability and clinical translational value of the interventions. Finally, due to the uneven geographic distribution of included studies—primarily from Asia and North America, with only one from Europe—the generalizability of our findings to Europe and other regions remains to be further validated.

## 6 Conclusion

This study suggests that mind–body exercise, as an adjunct intervention for breast cancer patients, shows promising potential in alleviating psychosocial distress. Pooled analyses indicate moderate and statistically significant positive effects on anxiety, depression, fatigue, and FCR. Although improvements in perceived stress, insomnia, and quality of life were also observed, the high heterogeneity led us to use a random-effects model to provide more conservative and generalizable effect estimates; nevertheless, the robustness and generalizability of these results remain limited and should be interpreted with caution. Meta-regression analyses indicated that intervention duration and regional factors were the main sources of heterogeneity for quality of life, while the sources of heterogeneity for perceived stress and insomnia could not be determined, suggesting that these outcomes may be influenced by other unmeasured variables.

In addition, mind–body exercise showed only marginal effects in reducing IL-6 levels, with limited evidence strength, and its effects on cognitive function, pain, and CRP levels were not clearly confirmed in this study. Overall, Mind–body exercise demonstrates promising short-term application value in the treatment of breast cancer patients, although its efficacy varies across different outcome measures, and long-term effects still need further validation. Future research should include rigorously designed, large-scale randomized controlled trials with extended follow-up periods, focusing on intervention type, duration, and target population characteristics, to provide more targeted and high-quality evidence for clinical practice.

## Data Availability

The original contributions presented in the study are included in the article/Supplementary material, further inquiries can be directed to the corresponding author.
