# Supplementary material for: The impact of mind–body exercise on female breast cancer patients—a systematic review and meta-analysis of randomized controlled trials
Source: Front Public Health. 2025 Oct 27;13:1641075. doi: 10.3389/fpubh.2025.1641075 (PMC12597735; doi:10.3389/fpubh.2025.1641075)
Supplement: Supplementary file 1 [file Data_Sheet_1.PDF]

Supplementary Material

3.5 Meta-regression results

Meta-regression Analysis of Anxiety Outcomes

	Coef.	Std. Err.	z	P>z	[95% Conf.	Interval]
Duration	.0614547	.0299152	2.05	0.040	.002822	.1200874
Region	.4845634	.4399173	1.10	0.271	-.3776586	1.346785
cancer stage	.1522395	.2287207	0.67	0.506	-.2960449	.6005239
Age	-.0494525	.0261135	-1.89	0.058	-.100634	.001729
Interventions	-.2312793	.1710313	-1.35	0.176	-.5664945	.103936
_cons	.9911788	1.258526	0.79	0.431	-1.475486	3.457844

Meta-regression Analysis of Depression Outcomes

	Coef.	Std. Err.	z	P>z	[95% Conf.	Interval]
Duration	-.0239482	.0212214	-1.13	0.259	-.0655414	.017645
Region	.5260213	.1550649	3.39	0.001	.2220995	.829943
Cancer stage	.0993695	.1270767	0.78	0.434	-.1496962	.3484352
Age	-.0336075	.0163923	-2.05	0.040	-.0657357	-.0014792
Interventions	.2733373	.1138706	2.40	0.016	.0501551	.4965195
_cons	.1461717	.7956576	0.18	0.854	-1.413288	1.705632

Meta-regression Analysis of QOL Outcomes

	Coef.	Std. Err.	z	P>z	[95% Conf.	Interval]
Duration	.1500286	.0531143	2.82	0.005	.0459265	.2541307
Region	-.9895949	.3425205	-2.89	0.004	-1.660923	-.3182671
Cancer stage	.1097816	.5921089	0.19	0.853	-1.050731	1.270294
Age	.0430858	.0329423	1.31	0.191	-.0214799	.1076516
Interventions	-.3443913	.1836992	-1.87	0.061	-.7044351	.0156525
_cons	-1.219499	1.738953	-0.70	0.483	-4.627785	2.188786

Note: QOL: Quality of Life.

Meta-regression Analysis of Fatigue Outcomes

	Coef.	Std. Err.	z	P>z	[95% Conf.	Interval]
Duration	.0162772	.0490723	0.33	0.740	-.0799027	.1124572
Region	.460999	.2651713	1.74	0.082	-.0587271	.9807251
Cancer stage	-.0601968	.1791371	-0.34	0.737	-.411299	.2909054
Age	.002925	.0335175	0.09	0.930	-.062768	.068618
Interventions	.1860983	.2125633	0.88	0.381	-.2305181	.6027146
_cons	-1.780269	1.643014	-1.08	0.279	-5.000518	1.43998

Meta-regression Analysis of Perceived Stress Outcomes						
	Coef.	Std. Err.	z	P>z	[95% Conf.	Interval]
Duration	.0781357	.1467881	0.53	0.595	-.2095636	.365835
Region	-.0942204	1.084019	-0.09	0.931	-2.218859	2.030418
Cancer stage	-.4786642	.4810414	-1.00	0.320	-1.421488	.4641597
Age	.0727098	.1107723	0.66	0.512	-.1443999	.2898196
Interventions	.0319198	.5544089	0.06	0.954	-1.054702	1.118541
_cons	-4.107477	5.608502	-0.73	0.464	-15.09994	6.884984

Meta-regression Analysis of FCR Outcomes						
	Coef.	Std. Err.	z	P>z	[95% Conf.	Interval]
Duration	-.0888137	.1893901	-0.47	0.639	-.4600115	.2823841
Region	(dropped)					
Cancer stage	(dropped)					
Age	.0449975	.0490246	0.92	0.359	-.051089	.141084
Interventions	(dropped)					
_cons	-2.351442	2.950967	-0.80	0.426	-8.135231	3.432348

Note: FCR: Fear of Cancer Recurrence.

Meta-regression Analysis of Insomnia Outcomes						
	Coef.	Std. Err.	z	P>z	[95% Conf.	Interval]
Duration	-.0449081	.1337505	-0.34	0.737	-.3070543	.2172381
Region	-.1388737	.7988511	-0.17	0.862	-1.704593	1.426846
Cancer stage	-.0212108	.4270091	-0.05	0.960	-.8581332	.8157116
Age	.068434	.0785427	0.87	0.384	-.0855068	.2223749
Interventions	.4685939	.4690257	1.00	0.318	-.4506795	1.387867
_cons	-4.259149	3.568589	-1.19	0.233	-11.25346	2.735158

Meta-regression Analysis of IL-6 Outcomes						
	Coef.	Std. Err.	z	P>z	[95% Conf.	Interval]
Duration	.0422835	.0319666	1.32	0.186	-.0203699	.1049368
Region	(dropped)					
Cancer stage	.4491742	.785585	0.57	0.567	-1.090544	1.988892
Age	-.0323363	.0258015	-1.25	0.210	-.0829064	.0182337
_cons	.3555407	2.09261	0.17	0.865	-3.7459	4.456981

Meta-regression Analysis of Pain Outcomes						
	Coef.	Std. Err.	z	P>z	[95% Conf.	Interval]
Duration	-.177825	.0630636	-2.82	0.005	-.3014274	-.0542226
Region	-.2240522	.2103058	-1.07	0.287	-.636244	.1881395
Cancer stage	-.198793	.168293	-1.18	0.238	-.5286412	.1310553
Age	.0463071	.0289985	1.60	0.110	-.0105289	.103143
Interventions	.880955	.3026205	2.91	0.004	.2878297	1.47408
_cons	-1.691319	1.328395	-1.27	0.203	-4.294926	.9122877

Meta-regression Analysis of Cognitive Function Outcomes						
	Coef.	Std. Err.	z	P>z	[95% Conf.	Interval]
Duration	-.2251908	.0835687	-2.69	0.007	-.3889824	-.0613992
Region	-2.764935	.8363027	-3.31	0.001	-4.404058	-1.125811
Age	.5062821	.1213636	4.17	0.000	.2684138	.7441503
_cons	-19.92168	4.669473	-4.27	0.000	-29.07368	-10.76968
